# Supplementary material for: FastqPuri: high-performance preprocessing of RNA-seq data
Source: BMC Bioinformatics. 2019 May 3;20:226. doi: 10.1186/s12859-019-2799-0 (PMC6500068; doi:10.1186/s12859-019-2799-0)
Supplement: Supplementary file 2 — Archive of FastqPuri. Archive containing all files needed to install and run FastqPuri v1.0.6. Date stamp March 22, 2019. (GZ 47,819 kb) [file 12859_2019_2799_MOESM2_ESM.gz › FastqPuri-1.0.6/latex/Qreport_8c__incl.pdf]

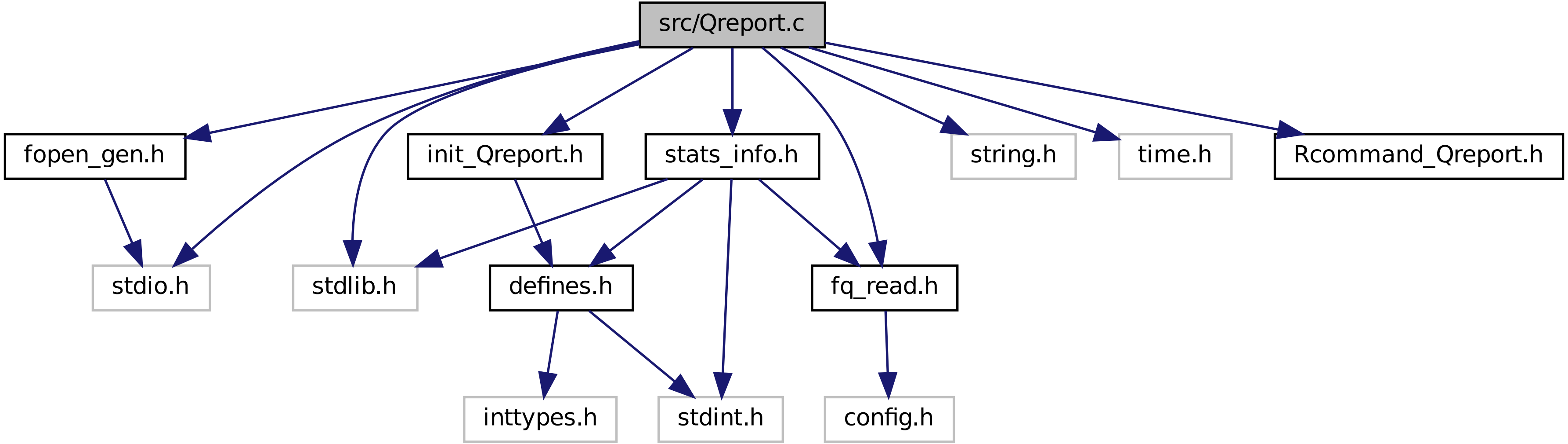
src/Qreport.c
fopen_gen.h
init_Qreport.h
stats_info.h
string.h
time.h
Rcommand_Qreport.h
stdio.h
stdlib.h
defines.h
fq_read.h
inttypes.h
stdint.h
config.h
